# Correction of aortic coarctation in a girl with severe PHACE syndrome

**DOI:** 10.1186/s13019-014-0169-6

**Published:** 2014-10-14

**Authors:** Lian Xiong, Zhenkun Xia, Chengming Fan, Weizhi Zhang, Jinfu Yang

**Affiliations:** Department of the cardiothoracic surgery, The 2nd Xiangya Hospital, Central South University, Middle renmin road 139, Changsha, 410011 China

**Keywords:** Vessel distortion, Aortic coarctation, Bypass, PHACE syndrome

## Abstract

A 12-year-old Chinese girl was demonstrated multivessel distortion and malformation: aortic coarctation with the narrowest lumen diameter measuring of 4 mm located between the left common carotid artery and the left subclavian artery, a huge and thin-walled aneurysm is connected to the coarctation, and the descending aorta was distorted. Cerebrovascular revealed distorted arteries and a completely aberrant brain blood supply. She underwent correction of the aortic coarctation by establishing a bypass between the ascending aorta and the descending aorta using a 13-mm Gore-tex tube. Postoperative recovery was uneventful, at 6-month follow-up, the cervical vascular pulsatility was relieved and she is in good condition.

**Electronic supplementary material:**

The online version of this article (doi:10.1186/s13019-014-0169-6) contains supplementary material, which is available to authorized users.

## Background

The PHACE acronym was coined in 1996 to describe the association of malformations of the posterior fossa, hemangiomas of the head and neck, arterial, cardiovascular, and eye anomalies, and ventral developmental defects [1],[2]. Cardiac and cerebrovascular anomalies are the most common extracutaneous features of PHACE, and they also constitute the greatest source of potential morbidity. Though the long-term outcomes of cardiovascular anomalies in PHACE remains undetermined, early surgical intervention are required [3]. Here we described the aortic and cerebral vasculopathy, and the successful surgical correction of aortic coarctation in a 12-year-old female with possible PHACE syndrome.

## Case presentation

A 12-year-old Chinese girl admitted with enhanced pulsatility of cervical artery, a heart murmur and a blood pressure gradient of 110 mmHg between upper and lower extremities. She was referred for aorta computed tomographic angiography (CTA) and aortogram for evaluation of her disease. Review of the image findings demonstrated an aortic coarctation with the narrowest lumen diameter measuring of 4 mm located between the left common carotid artery (LCCA) and the left subclavian artery (LSA), the descending aorta was distorted and the aorta section adjacent to maximal narrowing site was aneurismal (Figure 1). Cerebrovascular CTA revealed distorted arteries and a completely aberrant brain blood supply: the distal left internal carotid artery (LICA) and the origin of the right anterior cerebral artery (RACA) were vanished (Figure 2A). The basilar artery (BA) supplied blood for the whole left brain, the right posterior cerebral artery, and the RACA through the anterior communicating artery (Figure 2A). There was no cerebral aneurysm present, and she had no history of head symptoms. So correction of the aortic coarctation is urgent for her, a bypass between the ascending aorta and the descending aorta using a 13-mm Gore-tex tube (W.L. Gore & Associates, Inc, USA) was established under cardiopulmonary bypass (Figure 2B). Postoperative recovery was uneventful, and she was discharged on the 7th postoperative day fully recovered. At 6-month follow-up, the cervical vascular pulsatility was relieved, there is no postoperative blood pressure gradient between upper and lower extremities (upper limb BP: 120/72 mmHg; lower limb BP: 118/70 mmHg), and she is in good condition.

**Figure 1:**
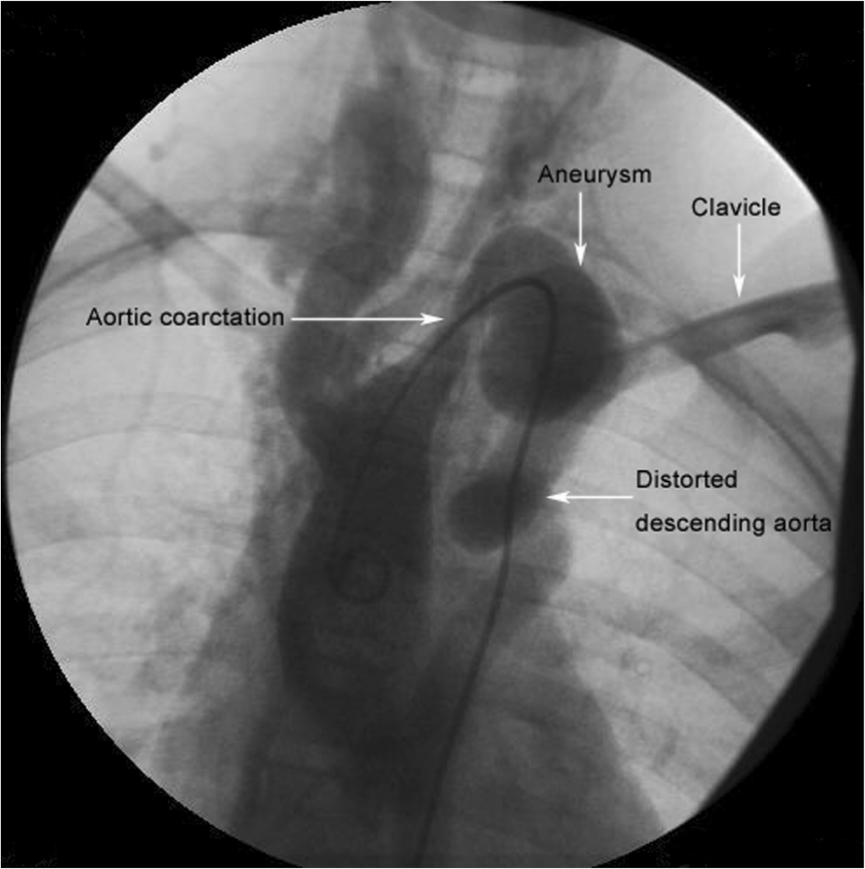
**Angiography image showed an aortic coarctation, aneurysm and the descending aorta distortion.** The white arrows mark the narrowest lumen located between the LCCA and the LSA, the aneurysm located in posterior mediastinum above the clavicle level, and the descending aorta is distorted.

**Figure 2:**
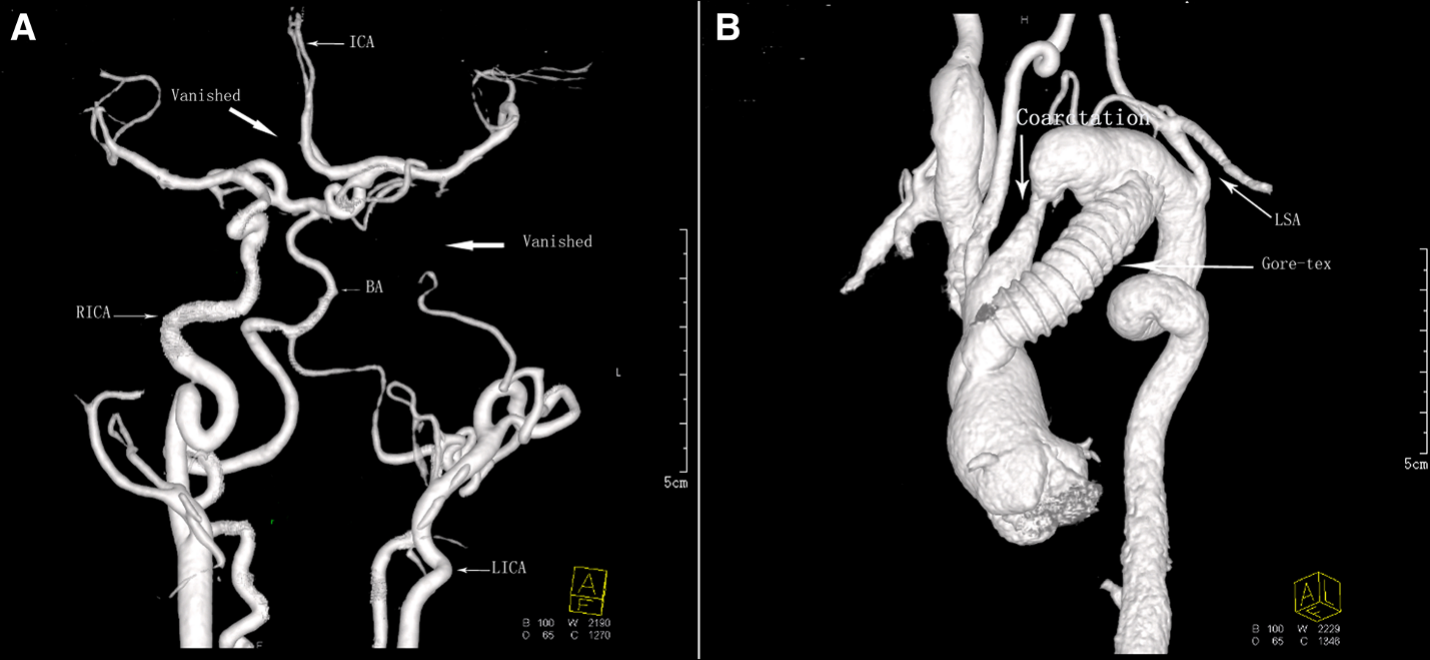
**CTA showed multivessel distortion and malformation. A**: Cerebrovascular CTA showed the distal LICA and the origin of the RACA were vanished, the BA supplied blood for the whole left brain. **B**: A 13-mm Gore-tex tube was implanted as a bypass between the ascending aorta and the descending aorta.

## Discussion

The combination of distortion of the large and middle sized arteries,aortic coarctation and cerebrovascular anomaly is an extremely rare event. Coarctation of aorta and tortuosity of the arteries throughout the body is often a feature of arterial tortuosity syndrome (ATS), which is characterized by tortuosity, stenosis, and aneurysm formation in the major arteries and SLCA10 gene mutation is the main pathogenesis for this disorder [4]. We detected the gene and found no mutation. Therefore taking her cardiovascular and cerebrovascular anomally into consideration, we speculated the girl presented PHACE syndrome, though the girl didn't have facial hemangiomas [5]. The most common and important anomaly of PHACE syndrome associated abnormalities are cerebrovascular and cardiovascular, which present in 91% and 67% of the patients respectively [6].

The surgical treatment for this disease is for stenosis and aneurysm. Although the descending aorta is distorted, the blood stream is not greatly affected and does not need operation. For this patient, we decided to perform an operation establishing a bypass between the ascending aorta and the descending aorta based on the following considerations: ? This patient's arch of aorta is shoved into posterior mediastinum and above the clavicle level by the dilated aneurysm, meanwhile a huge thin-wall aneurysm is formed immediately after the long narrow segment. Conventially, a patch plasty is used to correct constriction. However, in this case, despite the great difficulty of performing the operation, the risk of aneurysm rupture and massive hemorrhage is also tremendous. ? Blood vessel of 13 mm in diameter is sufficient for lower body circulation of a normal adult Chinese female [7], owing to there is still a little blood circulating in the patient's narrowed vessel (4 mm), which could increase the blood flow in distal part of coarctation. ? The patient is a 12-year-old Chinese female, whose circulation system is nearly fully developed. Hence, we decided to perform a bypass operation. The arch plasty has been wildly used as an approach to repair the aortic coarctation and the satisfactory surgical long term outcome has been reported by many surgeons from different centers in the world [8]. However, partial patients in some Chinese rural areas cannot afford the expensive medical costs because of some financial problem.

And therefore, instead of arch plasty, an alternative bypass procedure was also be used in some Chinese patients. And the long term surgical outcome for this relatively low-cost operation was reported by some Chinese surgeon [9]. In this case, the descending aorta diameter was (13?+?4) mm postoperation, so we did not think it is necessary for her to re-undergo the aortic operation in the future, because this aorta diameter meets the body size of a woman from the south part of China [7]. The advantages of bypass are minimal injury and relative safety, the 6-month follow-up shows that this bypass procedure is a satisfactory method for the treatment of PHACE syndrome. Of cause, a long term follow up is required.

However, the natural history and long-term prognosis of PHACES patients remains unknown. The cerebrovascular abnormalities are an important determinant of prognosis because of their propensity to cause acute ischemic events. Patients with PHACES who develop acute ischemic stroke usually present it before one year of age [10]. Aplasia, hypoplasia, or occlusion of a major cerebral artery appears to be a significant risk factor for arterial ischemic stroke in children with PHACE, especially when more than one vessel is involved or if there is coarctation of the aorta [11]. Thus earlier recognition and treatment of the disease might minimize the risk of neurologic disorders and a lifelong follow-up is needed.

## Conclusion

Earlier treatment of main malformation and lifelong follow-up for PHACES patient might obtain the ideal treatment results.

## Consent

Written informed consent was obtained from the patients or their legal guardians for publication of this case report. A copy of the written consent is available for review by the Editor-in-Chief of this journal.

## Authors' contributions

JY designed the operative procedure. JY, LX, ZX, WZ performed the surgical operations. CF drafted the manuscript. JY revised the manuscript. LX carried out the figure collection. All authors read and approved the final manuscript.

## Authors’ original submitted files for images

Below are the links to the authors’ original submitted files for images. Authors’ original file for figure 1 Authors’ original file for figure 2

## Abbreviations

CTA: Computed tomographic angiography
LCCA: Left common carotid artery
LSA: Left subclavian artery
LICA: Left internal carotid artery
RACA: Right anterior cerebral artery
BA: Basilar artery
