# Supplementary figures and images for: Correction of aortic coarctation in a girl with severe PHACE syndrome
Source: J Cardiothorac Surg. 2014 Oct 14;9:169. doi: 10.1186/s13019-014-0169-6 (PMC4203972; doi:10.1186/s13019-014-0169-6)

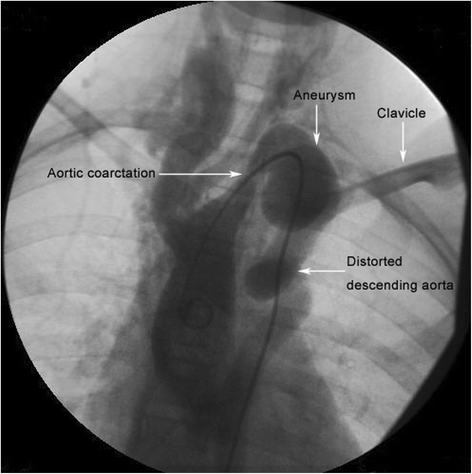

Supplement: Supplementary file 1 — Authors’ original file for figure 1 [file 13019_2014_169_MOESM1_ESM.gif]

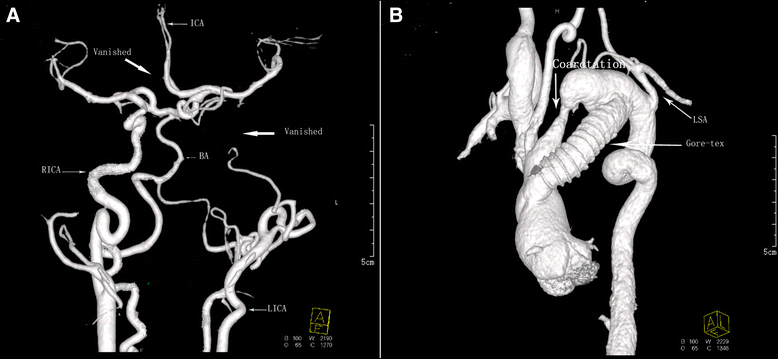

Supplement: Supplementary file 2 — Authors’ original file for figure 2 [file 13019_2014_169_MOESM2_ESM.gif]
